# CALiSol-23: Experimental electrolyte conductivity data for various Li-salts and solvent combinations

**DOI:** 10.1038/s41597-024-03575-8

**Published:** 2024-07-10

**Authors:** Paolo de Blasio, Jonas Elsborg, Tejs Vegge, Eibar Flores, Arghya Bhowmik

**Affiliations:** 1 Technical University of Denmark, Department of Energy Conversion and Storage, Kgs. Lyngby, 2800 Denmark; 2 SINTEF Industry, Sustainable Energy Technology, Trondheim, 7034 Norway

**Keywords:** Chemical physics, Batteries

## Abstract

Ion transport in non-aqueous electrolytes is crucial for high performance lithium-ion battery (LIB) development. The design of superior electrolytes requires extensive experimentation across the compositional space. To support data driven accelerated electrolyte discovery efforts, we curated and analyzed a large dataset covering a wide range of experimentally recorded ionic conductivities for various combinations of lithium salts, solvents, concentrations, and temperatures. The dataset is named as ’Conductivity Atlas for Lithium salts and Solvents’ (CALiSol-23). Comprehensive datasets are lacking but are critical to building chemistry agnostic machine learning models for conductivity as well as data driven electrolyte optimization tasks. CALiSol-23 was derived from an exhaustive review of literature concerning experimental non-aqueous electrolyte conductivity measurement. The final dataset consists of 13,825 individual data points from 27 different experimental articles, in total covering 38 solvents, a broad temperature range, and 14 lithium salts. CALiSol-23 can help expedite machine learning model development that can help in understanding the complexities of ion transport and streamlining the optimization of non-aqueous electrolyte mixtures.

## Background & Summary

Li-ion batteries (LIBs) are a cornerstone technology to enable the green transition, as they represent one of the most promising technologies for the storage of electrical energy generated from intermittent renewable sources to power the electrification of the electricity grid and transportation^1^. As LIBs charge and discharge, lithium ions diffuse and migrate through an electrolyte medium, shuttling between the battery electrodes that consume and produce them by electrochemical reactions at the electrode-electrolyte interfaces. The speed at which Li+ traverses the electrolyte is a crucial factor influencing the cycling capability of LIBs. Upon fast charge/discharge of the LIB, ions cannot diffuse and migrate fast enough to sustain the imposed cycling rate, and so they accumulate at the electrode interfaces, building concentration gradients that ultimately result in cell overpotentials and energy inefficiencies during the operation of LIBs^2,3^.

Given the critical role of ion transport in cell performance, electrolytes are typically designed to maximize their ionic conductivity. State-of-the-art LIB electrolytes consist of a Lithium salt dissolved in a liquid solution of multiple organic solvents mixed together. Salts must completely dissociate in the non-aqueous medium in order to maximize the number of Li+ available for transport, while the solvent mixture must provide a medium with high electric permittivity to facilitate dissolution of the salt, and low viscosity to facilitate ionic transport. Although ionic conductivity is a critical consideration, it is not the only property to optimize when designing electrolytes. Properties such as the temperature range, electrochemical stability, cost, toxicity, and flammability must all be considered to guarantee safe and long-lasting LIB operation. Only a small subset of electrolyte solutions comply with such strict requirements^4^.

As a result, designing new promising electrolytes is resource-intensive, as it involves not only experimental exploration of the composition space of salts and solvent mixtures but also testing each potential electrolyte against multiple desirable properties. The search for better electrolytes for conventional LIBs, could be greatly accelerated if ionic conductivity could be modeled and accurately predicted from electrolyte composition^5,6^. In this scenario, most experimental tests could be replaced by accurate model-based conductivity predictions across large compositional spaces, and instead reserved for only a few promising electrolyte candidates. Unfortunately, ion transport in concentrated liquid solutions is a highly complex process, for which no universal theory is available. Ionic transport is underpinned by electronic, coulombic, and steric molecular-level interactions, each influenced by the other and by salt concentration, temperature, and the physical-chemical properties of the individual molecules^7^. The development of accurate electrolyte models - whether ab initio^8^, thermodynamic^9,10^, empirical^11,12^, or data-driven^13–17^ - hinges upon the availability of high-quality experimental data for validation. As an inspiring case, the publication of large-scale battery cycling data^18,19^ has enabled the development of accurate models for battery lifetime prognosis with ever greater accuracy^20,21^. Data driven electrolyte conductivity models will play a crucial role in the rapid development of battery technology through multimodal workflows^6,22^.

In support of advancing the development of accurate electrolyte models, we present a curated dataset compiled from a comprehensive literature survey of non-aqueous electrolyte conductivity. Our digitization efforts have yielded the largest publicly available electrolyte conductivity data collection known to us. It covers a diverse range of 38 solvents, wide temperature ranges, and 14 different lithium salts. Each data point is expert ratified and rigorously referenced to its source publication, ensuring transparency and appropriate credit to the scientific work. We share this dataset with the scientific community, aiming to expedite the development and validation of electrolyte models, the understanding of ion transport complexities in concentrated liquid solutions, and ultimately to streamline the exploration of new, promising non-aqueous electrolytes.

## Methods

### Data Generation

**Fig. 1:**
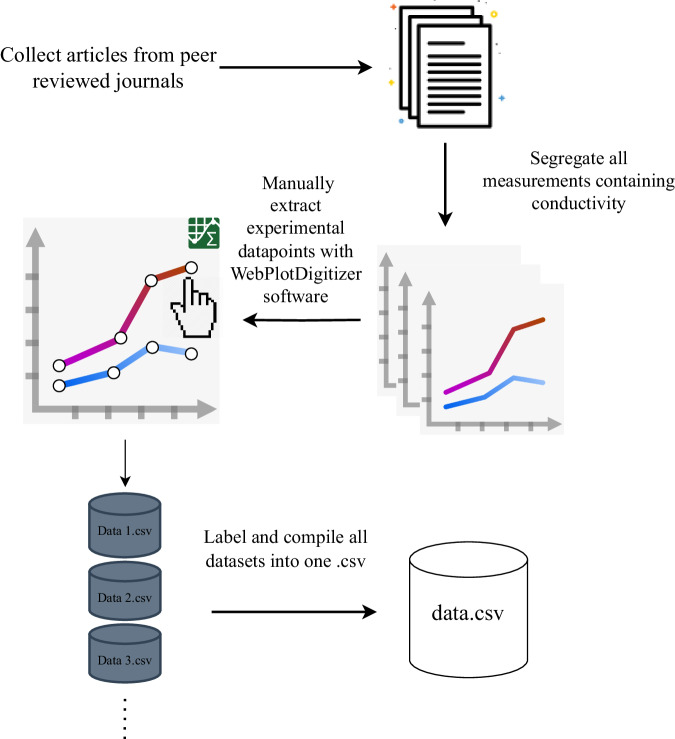
Workflow diagram of how the dataset was generated. We utilized Apps.Automeris WebPlotDigitizer^49^ - a software tool that enables manual data point extraction through the process of clicking on individual data points in a graph.

The data collection process is shown in Fig. 1, and can be outlined in two phases. Data acquisition was initialized by conducting an extensive literature search with Scopus, using the following search keywords:

- conductivity (Article title, Abstract, Keywords)
- AND electrolyte (Article title, Abstract, Keywords)
- AND lithium (Article title, Abstract, Keywords)
- AND (organic (Article title, Abstract, Keywords) OR non-aqueous (Article title, Abstract, Keywords))
- AND NOT polymer (Article title, Abstract, Keywords)
- AND NOT solid-state (Article title, Abstract, Keywords)
- AND NOT ionic liquid (Article title, Abstract, Keywords)

Out of the search results we selected the 200 most cited works, and selected articles with at least 10 conductivity measurements reported in a systematic series of experiments versus salt concentration, temperature or solvent mixture. Articles containing small datasets were not used for two main reasons. (A) To optimize human effort against dataset size. A significant part of the digitization time is spent on preparing the images and formatting the datapoints into our data model, it becomes impractical to digitize many small datasets. (B) To provide adequate data density in the dataset for machine learning tasks. A few observations of novel compositions (unless data from many articles each with few data point can be combined due to similarity in composition) contribute only marginally towards resolving the data manifold. Due to low data density in the corresponding part of chemical space Such data will likely add to the noise given that experimental conductivity measurements might have relatively high deviations and noise. As a result, we obtained 27 articles^12,23– 48^ that reported experimental data involving various organic solvents, lithium-ion salts, lithium salt concentrations, and temperatures. It is important to note that the present dataset is not immune to biases. It is known that conductivity values may vary according to the measurement method employed. In addition, human errors in experimental measurements and the limitations of our digitalization tools might all result in data imprecision. We have conducted validation procedures to detect to guarantee a basic level of consistency in the values, especially in measurements carried out on similar electrolytes and conditions but from multiple literature sources. Nevertheless, data users are encouraged to consult the individual publications to assess the quality of the measurements.

Subsequently, data was extracted from each selected paper by identifying and extracting all plots containing conductivity measurements at different temperatures, solvents, solvent ratios, lithium concentrations, and lithium salts. These graphical representations were obtained as image files and processed using specialized software, specifically the app Automeris WebPlot Digitizer 4.6^49^. This software allows for the extraction of data points from graphs through manual clicking on individual data points, resulting in a total of 13,825 experimentally measured data points.

The data points collected were then organized and structured into a .csv dataframe. The example conversions from weights to molar ratios were done using the Pandas^50,51^ and Numpy^52^ libraries. The workflow of the calculations and collection of data was built using Python 3.8.12. The combined CSV file was created using pandas. The plots were generated using the Matplotlib^53^ library.

## Data Records

**Fig. 2:**
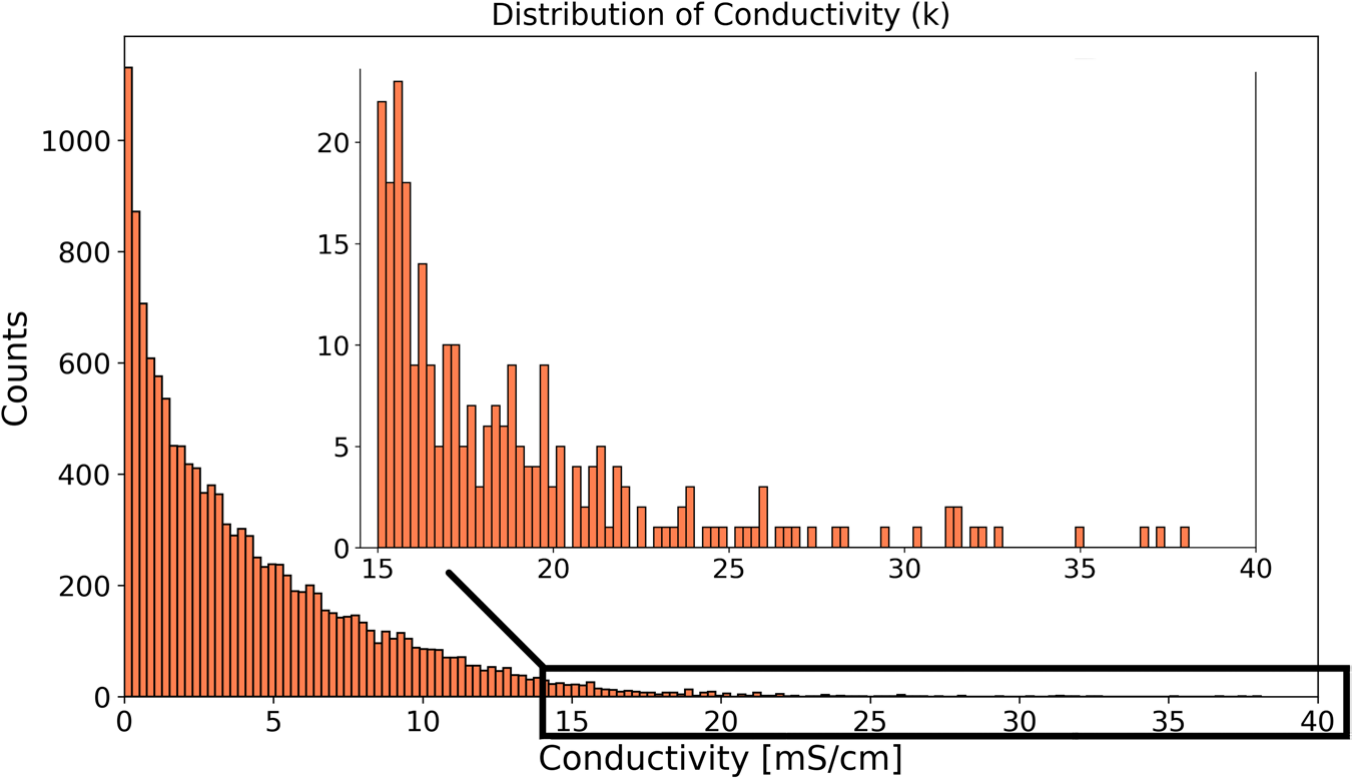
Distribution of conductivity *k* in *mS*/*cm* for the dataset with inset showing low-count values with *k* > 15. The figure shows that most measured conductivity values are close to the minimum value of 0, which can induce model biases towards fidelity at these values that are important to account for when constructing models based on the data.

CALiSol-23 is provided as a dataframe in a CSV file format, and can be downloaded from DTU Data^54^ under the entry name “CALiSol-23: Experimental electrolyte conductivity data for various Li-salts and solvent combinations”, and can be used under the CC BY license. Data were recorded for 27 different peer-reviewed academic journal articles and constitute 13,825 data points in total. Table 1 summarizes the data obtained from each academic article. The contents of each column in the data frame are summarized below, with the column name in parentheses:

- **DOI** (‘doi’) represents the Digital Object Identifier (DOI) for the article from which the data point was extracted, enabling the tracking of each point in the dataset. The DOIs in the CSV file correspond to the datasets contained in Refs. ^12,23–48^
- **Conductivity** (‘k’) represents the measured conductivity for a data point, such that every row reflects a measurement of a single conductivity reported as a floating point number. The values range from 0 mS/cm (Millisiemens per cm) to 38.1 mS/cm. Since this variable can be considered the primary dependent variable of interest, we show the distribution of values for this variable in Fig. 2.
- **Temperature** (‘T’) is the operating temperature under which the experiment corresponding to the data point was conducted, reported as a floating point number. The values range from 194.15 K (Kelvin) to 477.423 K.
- **Solvent Ratio Type** (‘solvent ratio type’) contains recorded strings that convey whether molar, volume, or weight ratio was used.
- **Concentration** (‘c’) represents the Lithium salt concentration, reported as a floating point number. The values range from 0 to 4.0, and are reported in units of either mol/kg (moles per kilogram) or mol/L (moles per liter), depending on the string recorded in the ‘c units’ column.
- **Lithium Salt Type** (‘salt’) is a string that represents the type of Lithium salt used in the experiment. Table 2 shows the formulas and chemical names of the salts present in the data.
- **Concentration Units** (‘c units’) represents the units in which the Lithium salt concentration was measured (mol/L or mol/kg).
- **Solvents** (‘EC’, ‘PC’, ‘DMC’, ‘EMC’, ‘DEC’, ‘DME’, ‘DMSO’, ‘AN’, ‘MOEMC’, ‘TFP’, ‘EA’, ‘MA’, ‘FEC’, ‘DOL’, ‘2-MeTHF’, ‘DMM’, ‘Freon 11’, ‘Methylene chloride’, ‘THF’, ‘Toluene’, ‘Sulfolane’, ‘2-Glyme’, ‘3-Glyme’, ‘4-Glyme’, ‘3-Me-2-Oxazolidinone’, ‘3-MeSulfolane’, ‘Ethyldiglyme’, ‘DMF’, ‘Ethylbenzene’, ‘Ethylmonoglyme’, ‘Benzene’, ‘g-Butyrolactone’, ‘Cumene’, ‘Propylsulfone’, ‘Pseudocumeme’, ‘TEOS’, ‘m-Xylene’, ‘o-Xylene’) correspond to single unique solvent types, such that the value of these represents the molar/volume/weight ratio (according to the value of the ‘solvent ratio type’ column) between the constituent solvents for that particular data point. Thus, the row values for these 38 columns for a single data point sum to 1. For data analysis purposes it might be convenient to convert all solvent ratios to molar ratios in order to obtain a consistent unit. In the GitHub repository, we have also made code available to perform this conversion^55^. Table 3 shows the formulas and chemical names of the solvents present in the data, as well as essential information on the data distributions associated with particular salt/solvent combinations.

**Table 1:** Summary of Electrolyte Properties for Li-ion Batteries: Ref., No. of Points, Salts, Solvents, Temperature Range (Min, Max), Rate Constant Range (Min, Max), and Salt Concentration Unit (mol/kg or mol/L).

Ref.	No. of Points	Salts	Solvents	T (Min, Max)	k (Min, Max)	Salt c Unit
^23^	2462	(LiBOB,)	(EC, PC, DEC)	(233.75, 332.15)	(0, 8.814)	mol/kg
^24^	1680	(LiPF6,)	(PC, DEC)	(194.15, 332.15)	(0.0, 13.31)	mol/kg
^25^	1630	(LiAsF6, LiN(CF3SO2)2, LiCF3SO3, LiPF6, LiBF4)	(‘EC’, ‘PC’, ‘DME’, ‘2-MeTHF’, ‘DMM’, ‘Freon 11’, ‘Methylene chloride’, ‘THF’, ‘Toluene’, ‘Sulfolane’, ‘2-Glyme’, ‘3-Glyme’, ‘4-Glyme’, ‘3-Me-2-Oxazolidinone’, ‘3-MeSulfolane’, ‘Ethyldiglyme’, ‘DMF’, ‘Ethylbenzene’, ‘Ethylmonoglyme’, ‘Benzene’, ‘g-Butyrolactone’, ‘Cumene’, ‘Propylsulfone’, ‘Pseudocumeme’, ‘TEOS’, ‘m-Xylene’, ‘o-Xylene’)	(213.15, 353.15)	(0.01, 38.1)	mol/L
^26^	1511	(LiBF4,)	(EC, PC, DEC)	(233.75, 332.15)	(0, 8.679)	mol/kg
^27^	1268	(LiBF4,)	(PC, DEC)	(233.75, 332.15)	(0, 6.652)	mol/kg
^28^	1245	(LiPF6,)	(EC, PC, DEC)	(233.75, 332.15)	(0.016, 13.908)	mol/kg
^29^	810	(LiBOB,)	(PC, EA)	(233.75, 332.15)	(0.166, 17.154)	mol/kg
^30^	651	(LiPF6,)	(EC, PC, EMC, TFP)	(243.15, 333.15)	(0, 16.242)	mol/kg
^31^	616	(LiBOB, LiBPFPB, LiBMB, LiBPFPB)	(PC, DME, DMSO)	(203.038, 477.423)	(0.0, 22.97)	mol/L
^32^	416	(LiBOB, LiCF3SO3, LiClO4, LiTFSI, LiBPFPB, LiBMB)	(PC, DME, DMSO, AN)	(207.21, 393.642)	(0.002, 37.361)	mol/L
^33^	325	(LiPF6,)	(EC, EMC)	(233.15, 333.15)	(0.314, 16.465)	mol/kg
^34^	240	(LiPF6, LiBF4)	(PC, DEC)	(233.15, 293.15)	(0.0, 6.756)	mol/kg
^35^	169	(LiPF6,)	(EC, DMC, EMC, MA)	(273.033, 313.15)	(3.259, 20.278)	mol/kg
^36^	160	(LiPF6,)	(EC, DMC, EMC)	(273.15, 313.15)	(1.12, 15.35)	mol/kg
^37^	131	(LiTFSI, LiPF6)	(EC, DMC)	(256.115, 354.095)	(3.38, 22.553)	mol/L
^12^	119	(LiPF6,)	(EC, DMC, EMC, FEC)	(263.15, 323.15)	(0.058, 17.36)	mol/L
^38^	110	(LiClO4, LiPF6)	(EC, 2-Glyme)	(233.038, 328.15)	(0, 16.435)	mol/L
^39^	49	(LiBF4, LiFSI, LiPF6)	(EC, EMC)	(253.14, 332.812)	(1.294, 15.686)	mol/L
^40^	45	(LiAsF6,)	(PC, DME)	(248.15, 298.15)	(0.208, 1.739)	mol/kg
^41^	39	(LiTDI, LiPDI, LiPF6)	(EC, DMC)	(253.424, 317.945)	(1.842, 16.979)	mol/L
^42^	32	(LiPF6,)	(EC, PC, DMC)	(263.0, 333.0)	(0.0, 19.634)	mol/L
^43^	29	(LiPF6,)	(MOEMC,)	(212.907, 343.391)	(0.003, 5.668)	mol/L
^44^	24	(LiPF6, LiBF4)	(EC, EMC)	(222.446, 333.15)	(0.13, 14.058)	mol/L
^45^	23	(LiPF6, LiBF4)	(EC, DMC, DEC)	(223.497, 333.235)	(0.025, 0.161)	mol/kg
^46^	18	(LiCF3SO3, LiTFSI, LiClO4)	(DME, DOL, 4-Glyme)	(298.0, 298.0)	(1.324, 10.968)	mol/L
^47^	15	(LiPF6,)	(EC, DEC)	(283.15, 313.15)	(3.786, 10.643)	mol/L
^48^	8	(LiPF6,)	(EC, EMC)	(298.15, 298.15)	(4.534, 9.507)	mol/L

## Technical Validation

This work aims to provide a comprehensive dataset that can be used to analyze the relationship between the conductivity of different Lithium salt types in various solvents and operating conditions. To comprehensively address the need for a better understanding of lithium-ion transport in organic solvents, other characteristics such as density, viscosity, and electrochemical stability should be recorded. We suggest that future experimental work keeps a complete data record (i.e. includes data beyond what is needed to address the primary research question of the concerned publication if available) such that the reusability of said data is increased. However, we believe that the present dataset will be helpful to resolve challenges involved in establishing relationships between electrolyte properties and conductivity.

**Table 2:** Salt Formulas and Chemical Names.

Salt Formula	Chemical Name
LiPF6	Lithium hexafluorophosphate
LiBF4	Lithium tetrafluoroborate
LiFSI	Lithium Bis(fluorosulfonyl)imide
LiTDI	Lithium 2-trifluoromethyl-4,5-dicyanoimidazole
LiPDI	Lithium 4,5-dicyano-2-(pentafluoroethyl)imidazolide
LiTFSI	Lithium bis(trifluoromethanesulfonyl)imide
LiClO4	Lithium perchlorate
LiAsF6	Lithium hexafluoroarsenate(V)
LiBOB	Lithium bis(oxalato)borate
LiCF3SO3	Lithium triflate
LiBPFPB	Lithium bis(perfluoropinacolato)borate
LiBMB	Lithium bis(malonato)borate
LiN(CF3SO2)2	Lithium bis(trifluoromethanesulfonimide)

**Table 3:** Solvent Names, Column Names, and Formulas.

Solvent Name	Column Name	Formula
Ethylene carbonate	EC	${({\mathrm{CH}}_{2}{\rm{O}})}_{2}\mathrm{CO}$
Propylene carbonate	PC	C_4_H_6_O_3_
Dimethyl carbonate	DMC	$\mathrm{OC}{({\mathrm{OCH}}_{3})}_{2}$
Ethyl Methyl Carbonate	EMC	C_4_H_8_O_3_
Diethyl carbonate	DEC	$\mathrm{OC}{({\mathrm{OCH}}_{2}{\mathrm{CH}}_{3})}_{2}$
Dimethoxyethane	DME	C_4_H_10_O_2_
Dimethyl sulfoxide	DMSO	C_2_H_6_OS
Acetonitrile	AN	CH_3_CN
2-Methoxyethyl (methyl) carbonate	MOEMC	C_5_H_10_O_4_
Tris(2,2,2-trifluoroethyl) phosphate	TFP	C_6_H_6_F_9_O_4_P
Ethyl acetate	EA	CH_3_CO_2_CH_2_CH_3_
Methyl acetate	MA	CH_3_COOCH_3_
Fluoroethylene carbonate	FEC	C_3_H_3_FO_3_
Dioxolane	DOL	${({\mathrm{CH}}_{2})}_{2}{{\rm{O}}}_{2}{\mathrm{CH}}_{2}$
2-Methyltetrahydrofuran	2-MeTHF	C_5_H_10_O
Dipropylene glycol dimethyl ether	DMM	C_7_H_16_O_3_
Trichlorofluoromethane	Freon 11	CCl_3_F
Methylene chloride	Methylene chloride	CH_2_Cl_2_
Tetrahydrofuran	THF	C_4_H_8_O
Toluene	Toluene	C_7_H_8_
Sulfolane	Sulfolane	${({\mathrm{CH}}_{2})}_{4}{\mathrm{SO}}_{2}$
Diglyme	2-Glyme	${({\mathrm{CH}}_{3}{\mathrm{OCH}}_{2}{\mathrm{CH}}_{2})}_{2}{\rm{O}}$
Triglyme	3-Glyme	C_8_H_18_O_4_
Tetraglyme	4-Glyme	C_10_H_22_O_5_
3-Me-2-Oxazolidinone	3-Me-2-Oxazolidinone	C_4_H_7_NO_2_
3-Methylsulfolane	3-MeSulfolane	C_5_H_10_O_2_S
2-(2-Ethoxyethoxy)ethanol	Ethyldiglyme	CH_3_CH_2_OCH_2_CH_2_OCH_2_CH_2_OH
Dimethylformamide	DMF	${({\mathrm{CH}}_{3})}_{2}\mathrm{NC(O)H}$
Ethylbenzene	Ethylbenzene	C_6_H_5_CH_2_CH_3_
Ethylene glycol monomethyl ether	Ethylmonoglyme	C_3_H_8_O_2_
Benzene	Benzene	C_6_H_6_
gamma-Butyrolactone	g-Butyrolactone	C_4_H_6_O_2_
Cumene	Cumene	C_9_H_12_
Propyl Sulfone	Propylsulfone	C_6_H_14_O_2_S
1,2,4-Trimethylbenzene	Pseudocumeme	${{\rm{C}}}_{6}{{\rm{H}}}_{3}{({\mathrm{CH}}_{3})}_{3}$
Tetraethyl orthosilicate	TEOS	$\mathrm{Si}{({\mathrm{OC}}_{2}{{\rm{H}}}_{5})}_{4}$
m-Xylene	m-Xylene	${{\rm{C}}}_{6}{{\rm{H}}}_{4}{({\mathrm{CH}}_{3})}_{2}$
o-Xylene	o-Xylene	${{\rm{C}}}_{6}{{\rm{H}}}_{4}{({\mathrm{CH}}_{3})}_{2}$

To document the breadth of the dataset, we present the distribution of data points from measured conductivities in Fig. 2. An exponentially decreasing distribution is observed, with ~ 95% of recorded data points having a conductivity below 15 *mS* *cm*^−1^. For this reason, we show both the distribution of all recorded conductivities in Fig. 2 as well as an inset of the distribution of conductivities for *k* > 15 *mS* *cm*^−1^. Figure 2 can also be considered a representative snapshot that effectively captures the range of values of conductivity on non-aqueous Li-ion electrolytes, and as such it enables straightforward comparisons with conductivity values across other emergent electrolyte technologies such as solid-state electrolytes and ionic liquids.

Most data points were collected at temperatures between 230 − 330*K*, i.e. between − 43^°^*C* and 57^°^*C*, which are typical temperature ranges for the operation of Li-ion batteries^56^. In the data, solvent ratios were recorded in weight or volume for more than 90 % of the data points. Around 77% of data points had salt concentration, *c*, recorded in molality (*mol*/*kg*), while the rest had salt concentration recorded in molarity (*mol*/*L*). Unlike solvent ratio units for which there is a straightforward conversion, the concentration units of molarity and molality cannot be interconverted unless the density of the solution is known; in most studies, such density is not reported. Therefore, aggregating measurements carried out in *mol*/*kg* and *mol*/*L* would require experimental determination of the density of the electrolyte solution.

In total, 14 Lithium salt types are present in the final dataset. Of these, ~ 93 % of data was extraced for the salts LiPF_6_, LiBF_4_, LiAsF_6_ and LiBOB. The remaining 10 salt types all have less than 300 counts. We refer to Table 1 for an overview of the data contained in CALiSol-23.

**Fig. 3:**
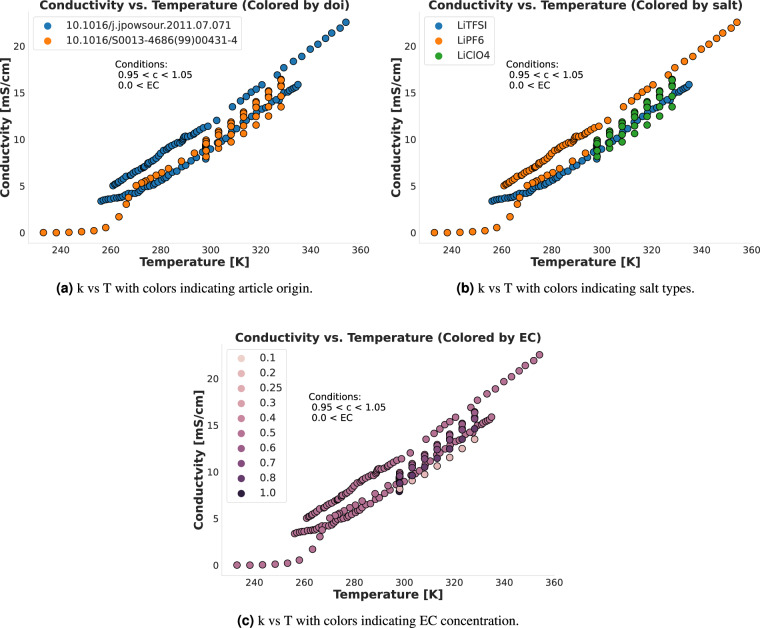
Comparing the T vs k relationship for data points with a concentration close to 1 that used EC as a solvent. The three panels show that similar concentrations and salt types behave similarly, even when taken from different sources, and thereby demonstrate the technical coherence of CALiSol-23 even though the data originates from multiple sources.

**Fig. 4:**
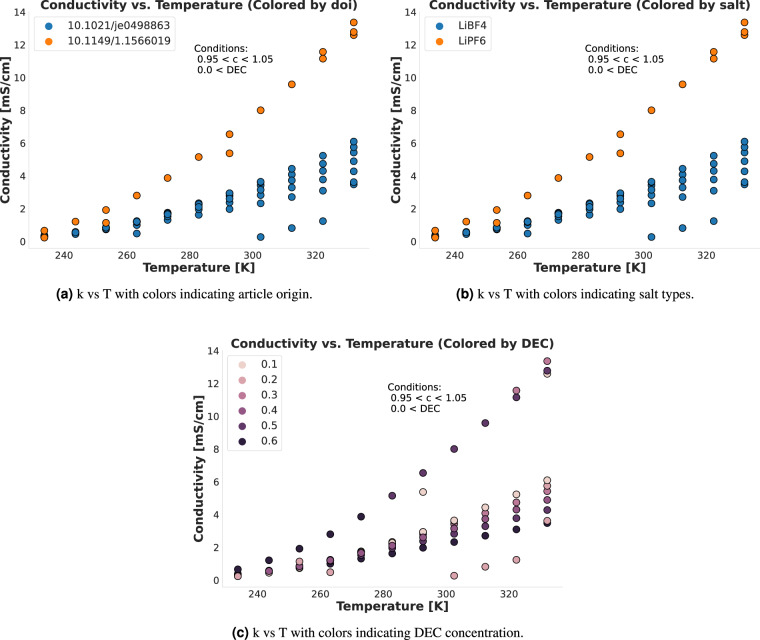
Comparing the T vs k relationship for data points with a concentration close to 1 that used DEC as a solvent. As with Fig. 3, the three panels show that data points with similar concentrations and salt types from different sources behave similarly.

Given that the dataset is derived from various experimental sources, the consistency of data must be assessed, which can be done by analyzing data from different sources under similar conditions. The accuracy of the measurement is quantified by the spread of possible values obtained with multiple observations due to systemic bias coming from the limitations in the measurement process. Such limitations stem mainly from instrumental accuracy but can also depend on the material composition. To assess the consistency of data across different sources, we generated plots that show how data varies in two subsets of the full dataset in Figs. 3 and 4. These plots showcase the behavior of data from several sources under similar concentrations and with similar solvents. The importance of this analysis lies in its capacity to confirm the reproducibility and reliability of data across different experiments. The observed alignment of data trends from diverse sources underlines the reliability of the dataset. This ensures that the dataset is not solely reflective of peculiarities specific to sources or experimental setups but captures the trends originating from physico-chemical phenomena present in non-aqueous electrolyte transport. For example, in Fig. 4a,b, it is evident that data points retrieved using the LiPF_6_ salt show similar temperature-conductivity behavior in two sources, and is qualitatively different than that of LiBF_4_, which was retrieved from four other sources.

**Fig. 5:**
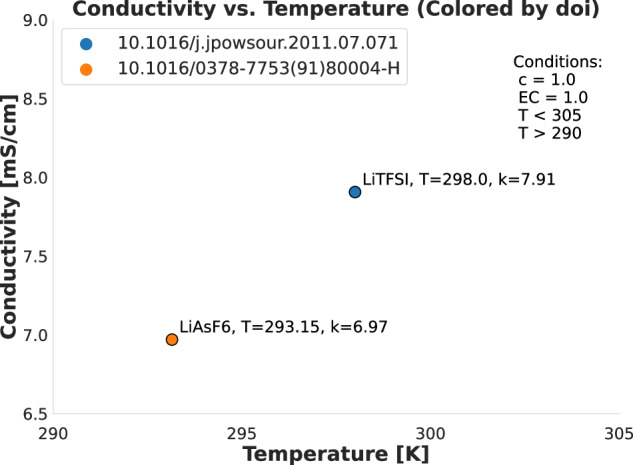
Two similar data points taken from the larger collection of points using the same conditions as in Fig. 3, stemming from two different sources. The main difference is the salt type, and thus the figure shows that similar conditions will differ when salt types differ.

**Fig. 6:**
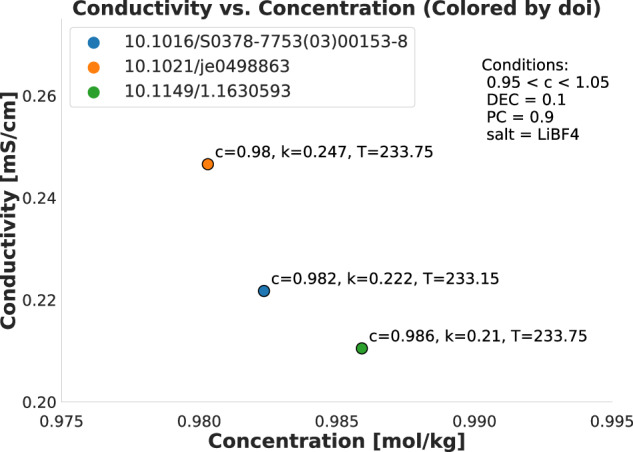
Three similar data points taken from the larger collection of points using the same conditions as in Fig. 4, stemming from three different sources. All three points have the same salt type and solvents but differ slightly in concentration, which leads to discernible differences in the recorded conductivities.

To supplement Figs. 3 and 4, we also produced Figs. 5 and 6. These figures show a minimal set of very similar data points to show the (dis)similarities between them. Figure 5 shows two data points from different sources that are also present in Fig. 3, where the only major difference is that one uses the LiTFSI salt and the other uses the LiAsF_6_ salt, although a slight temperature difference is also present. The plot thus shows that the general (T, k) neighborhood is similar for the two sources with very similar conditions, although the salt makes a noticeable difference. This validates that we can reliably compare data from different sources. Similarly, Fig. 6 shows three data points also present in Fig. 4. These data points are fully equivalent in terms of the specific solvents used (DEC at a relative concentration of 0.1 and PC at a relative concentration of 0.9), the Lithium salt (LiBF_4_) and temperature *T* ≈ 233*K*. The plot fully demonstrates that all three points are in very close proximity to each other on the (c, k) curve, with a decreasing trend in *k* as *c* increases that can be followed across the three points even though they stem from three different sources. Thus, the combined effect of Figs. 3–6 demonstrates and technically validates the coherence of CALiSol-23.

## Usage Notes

This dataset can be used for building models that maps component concentrations (of a fixed set of constituents) to temperature dependent ionic conductivities^6,13,57^. However, a more interesting utilization would be in the development of models that unify the molecular structure representation with the concentrations to enable screening for compositions with constituent molecules that are beyond the dataset. However, the validity of the model in the neighbourhood chemical space of molecules present in the dataset, will depend on the continuity and smoothness of the representation function in the chemical space. Identification of such appropriate descriptors requires exploring^58,59^ a broad range machine learned and cheminformatics based representations^60,61^ in combination with a wide variety of predictive classical^62,63^ and machine learning models^57,64–68^ and performing exhaustive testing. Molecular representations used by these models from the provided SMILES strings or after SMILES those to other datatypes like InChi, atomic graphs, or atomic position-based descriptions using cheminformatics tools like RDKit.

All the data are publicly available from DTU Data^54^, under the entry name “CALiSol-23: Experimental electrolyte conductivity data for various Li-salts and solvent combinations”, as a single CSV file under the CC BY 4.0 license. Scripts for visualizing data distributions are available from the GitHub repository under the MIT license condition.

## Data Availability

All scripts for summarizing and visualizing the data are available on GitHub under the MIT license agreement^55^.
